# Human MLPA Probe Design (H-MAPD): a probe design tool for both electrophoresis-based and bead-coupled human multiplex ligation-dependent probe amplification assays

**DOI:** 10.1186/1471-2164-9-407

**Published:** 2008-09-10

**Authors:** Jizu Zhi, Eli Hatchwell

**Affiliations:** 1 Genomics Core Facility, School of Medicine, Stony Brook University, Stony Brook, NY 11794, USA

## Abstract

**Background:**

Multiplex ligation-dependent probe amplification (MLPA) is an efficient and reliable technique for gene dosage analysis. Currently MLPA can be conducted on two platforms: traditional electrophoresis-based, and FlexMAP bead-coupled. Since its introduction in 2002, MLPA has been rapidly adopted in both clinical and research situations. However, MLPA probe design is a time consuming process requiring many steps that address multiple criteria. There exist only one or two commercial software packages for traditional electrophoresis-based MLPA probe design. To our knowledge, no software is yet available that performs bead-coupled MLPA probe design.

**Results:**

We have developed H-MAPD, a web-based tool that automates the generation and selection of probes for human genomic MLPA. The software performs physical-chemical property tests using UNAFold software, and uniqueness tests using the UCSC genome browser. H-MAPD supports both traditional electrophoresis-based assays, as well as FlexMAP bead-coupled MLPA.

**Conclusion:**

H-MAPD greatly reduces the efforts for human genomic MLPA probe design. The software is written in Perl-CGI, hosted on a Linux server, and is freely available to non-commercial users.

## Background

Multiplex ligation-dependent probe amplification (MLPA) has proven to be an efficient and reliable technique for gene dosage analysis. This technique was first introduced in 2002 [1], and has been widely applied in a variety of clinical and research situations. In this method, two sequence-tagged half probes are annealed to adjacent sites on the genomic target sequence and ligated using a thermostable DNA ligase. The ligated probes are subsequently amplified with universal PCR primers (one of which is fluorescently labeled) and quantified using electrophoresis (each product has a distinct size, which allows for identification) (See additional file 1: Diagram of electrophoresis-base MLPA). A typical, capillary-based MLPA assay allows for the quantification of up to 45 distinct sequences. In a more recent development, FlexMAP bead-coupled MLPA has been described, which allows for the simultaneous detection of 100 distinct sequences per reaction (identification of distinct sequences, which can all be the same size, is based on association with a distinct bead) [2] (See additional file 2: Diagram of bead-coupled MLPA).

Compared to other gene dosage detection methods, including, for example, Southern blot analysis and FISH (fluorescent in situ hybridization), MLPA is high throughput, requires only small amounts of starting DNA (cf. Southern blotting), does not require cells for chromosome spreads (as in FISH) and can be used to target any genomic sequences for copy number analysis, irrespective of their size or proximity to each other. Quantitative PCR (qPCR) is a potential alternative, but its use in a multiplex assay is limited by the spectral overlap of the fluorescent dyes used [1], and detecting 2:1 or 3:2 copy number change is challenging.

However, while kits covering multiple regions/genes of interest are available from MRC-Holland, for those regions not yet commercially available as kits, there are significant challenges in designing custom assays for MLPA. In the original description, MLPA probe sets were generated by cloning the target specific sequence into a family of M13 derived vectors that already contained the variable length fragments. With improvements in oligonucleotide synthesis, completely synthetic probe sets have been successfully used for MLPA reactions, obviating the need for the implementation of the M13 method [3]. The current major obstacle to successful MLPA probe design is the long list of criteria that need to be simultaneously satisfied to improve the likelihood of a successful assay. These criteria include probe length, Tm, secondary structure, GC content, nucleotide composition at the ligation site, sequence uniqueness, avoidance of known SNPs, etc. Manual probe design is time consuming and error prone. Our software automates this tedious MLPA probe design process.

## Implementation and results

### Input

H-MAPD accepts one or more nucleotide sequences in FASTA format. Due to technical limitations, electrophoresis-based MLPA supports up to 45 assays per reaction (per fluorescent reporter), while bead-coupled MLPA currently supports up to 100 assays per reaction. The software sets a maximum limit of 50 and 100 sequences for electrophoresis-based and bead-coupled MLPA, respectively. To prevent server overloading, aggregate sequence lengths up to 100,000 bases are allowed (equal to 100 sequences with an average length of 1000 nucleotides). Two platforms are provided, depending on the available technology: one for traditional electrophoresis-based and one for FlexMap bead-coupled MLPA.

### Probe generation

For the electrophoresis-based MLPA platform, the probe length is increased by 4 bases for each input sequence, starting with the minimum length of ligation product specified by the user. If the user chooses to use stuffer sequences (See additional file 3: Stuffer sequences), the probe length increment is achieved by adding stuffer sequences between primer and hybridizing sequences in both the left (LPO) and right probe oligos (RPO). Otherwise, the increment is achieved by extending the length of the hybridizing sequences. For bead-coupled MLPA, all probes have the same length. The tag sequences included in the probe for each bead are provided in additional file 4: FlexMAP bead tag sequences (these are complementary to the anti-tag sequences that are provided as pre-synthesized bead-coupled oligos).

For each input sequence, the length of the hybridizing sequence (left and right combined) is calculated by subtracting the length of primers, stuffers or bead tag sequence from the total probe length. A series of hybridizing sequences are generated by walking along the input sequence at 1 base steps, and extracting fragments of the desired length. The hybridizing sequences are split in the middle: the left hybridizing sequence (LHS) becomes the 3' end of the LPO and the right hybridizing sequence (RHS) becomes the 5' end of the RPO.

### Probe screening

**Figure 1:**
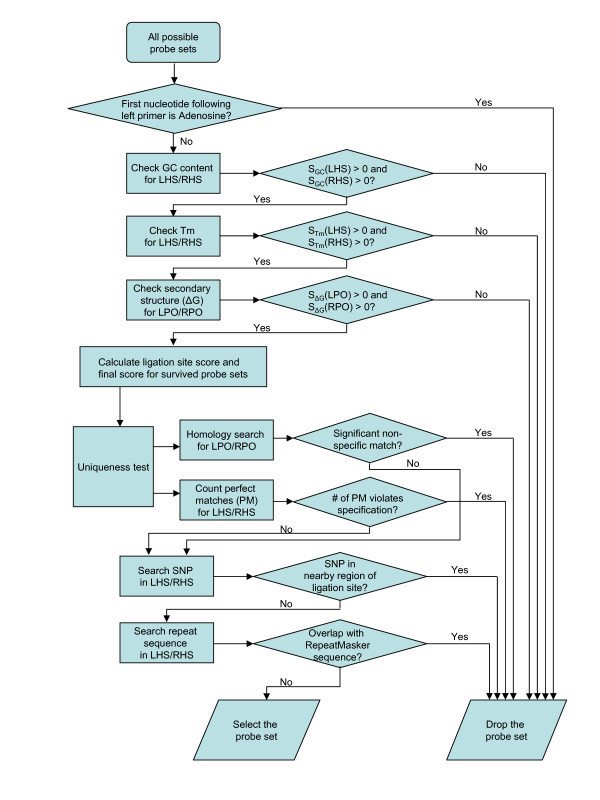
**Outline of probe selection workflow**. A successful probe set needs to meet all the criteria specified in the diagram.

**Figure 2:**
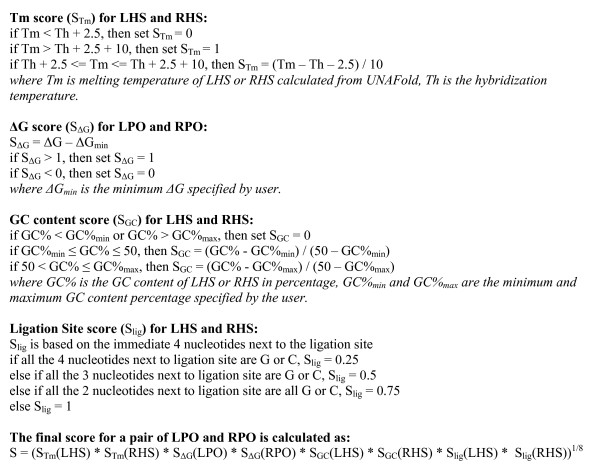
**Score calculation for probe sets**. The final score for each probe set is determined by the scores of Tm, ΔG, GC content and ligation site. Since each individual score falls in the range [0, 1], the final score should also fall in range [0, 1], with 1 being the best score. Probe sets with a final score > 0 are processed for further tests.

MRC-Holland has the most experience in MLPA probe design. H-MAPD adopted most of the criteria described in MRC-Holland's probe design guidelines [4]. The workflow of probe selection is outlined in Figure 1. It has been observed that the first nucleotide following the left primer sequence affects probe signal strength [1], and it is suggested that adenosine should be avoided at this position [4]. H-MAPD has followed this suggestion and excludes adenosine at the first position following the left primer in the LPO. Empirically, the ideal GC content of hybridizing sequence is around 50% [3,4], however a GC content of 28% has been successfully applied in MLPA [3]. Our software allows users to specify different ranges of GC content for the LHS and RHS, but ranges close to 50%, for example, 40–60% and 35–65% are recommended. The Tm is very important when considering how hybridizing sequences anneal to template DNA. Accurate Tm prediction for oligonucleotides is complicated and different algorithms result in different Tm values. MRC-Holland uses RAW for Tm prediction. While RAW runs only on the Windows operating system, H-MAPD calculates Tm using UNAFold software that supports various operating systems [5]. Additional file 5: Comparison of Tm calculated by RAW and UNAFold, compares melting temperatures calculated using RAW and UNAFold for the reference sequences mentioned in the MRC-Holland MLPA probe design guidelines [4]. Tm calculated by RAW is on average 9.1°C higher than that calculated by UNAFold with a standard deviation of 2.8°C. MRC-Holland recommends a minimum Tm (calculated by RAW) of 8°C above hybridization temperature. H-MAPD ensures both the LHS and RHS should have a Tm (calculated by UNAFold) of 2.5°C above hybridization temperature. Secondary structure prediction is performed on the LPO and RPO, also using UNAFold software. Both LPO and RPO should pass a minimum threshold for ΔG. Although a ΔG ≥ 0 is preferred, many probes having a negative ΔG actually work [4], therefore the software allows the user to choose a negative ΔG. Finally, to ensure efficient ligation, it is recommended that the sequences immediately adjacent to the ligation site should contain no more than three G and/or C [4]. H-MAPD favours low GC content at the ligation site by assigning a low ligation site score to high GC occurrence. The final score calculation for a probe set is shown in Figure 2.

Probe sets that meet all physical-chemical criteria (with a final score > 0) are subject to uniqueness screening. Homology search is performed for LPO and RPO using our local partial mirror of the UCSC BLAT server [6,7]. Since hybridizing sequence is from genomic DNA, in order to avoid interference from pseudogenes or closely related genes, both LHS and RHS should have one and only one perfect match in the same region of the genomic DNA. However, it is conceivable that a user would try to design MLPA probes for a region which has multiple copies in the reference genome assembly. In order to allow this possibility, users can specify the maximum number perfect matches for the full hybridizing sequence (LHS + RHS) in the reference genome. The Tm is calculated for all other non-specific (undesirable) matches. If the Tm is above (hybridization temperature – 5.0)°C, the probe set is dropped. This ensures that the Tm of LHS and RHS is at least 7.5°C above any non-specific matches. Next, a SNP search is performed using the latest SNP database (snp128 at time of writing) included in the UCSC BLAT server. In the Stuffer and Bead protocols, LHS and RHS are usually not long. In the No-Stuffer protocol, LHS and RHS can often reach more than 100 nucleotides in length. For short LHS/RHS (less than 40 nucleotides), if any SNP is detected anywhere in the LHS or RHS, the probe set is dropped. For LHS/RHS longer than 40 nucleotides, only the adjacent 40 nucleotides on each side of the ligation site are tested for SNP occurrence. SNP(s) that are located 40 nucleotides away from the ligation site should not affect annealing and ligation of hybridizing sequences. Finally, repeat sequences are more prone to cause non-specific binding due to their abundance in the genome. Therefore, if either LHS or RHS overlaps with regions defined in the UCSC genome browser RepeatMasker track, an extra criterion (Maximum repeat sequence match allowed) is applied.

### Output

Probe sets passing all criteria will be sorted by their scores and returned online (a link will be sent via email to the user upon completion of the analysis). Depending on the size of the input sequence, results will be returned in minutes or hours.

## Discussion

Our software applies to human genomic MLPA probe design only but will be generalized in the future to deal with other genomes. In addition, H-MAPD is currently only for the design of MLPA copy number assays, but may be generalized in the future to deal with the design of MLPA assays for other applications, such as SNP detection and methylation changes. For multiple input sequences, the stuffer and bead tag modifications will be applied to the input sequences sequentially. For example, in bead-coupled MLPA, the first bead tag will be inserted into the first input sequence and so on.

The software takes into consideration most factors predicted to affect MLPA probe performance. However, there are likely to be unexpected factors that might cause problems. These factors include secondary structure of genomic DNA at the hybridization site, the presence of un-described SNP(s) at the hybridization site, sequence errors in the reference genome assembly, etc. The software performs sequence homology searches using BLAT due to its speed and excellent programming interface. Another popular sequence search tool is BLAST. Because they are implemented using different algorithms, BLAT and BLAST may not return identical results. The authors strongly recommend that users verify H-MAPD results with BLAST for independent validation.

Two platforms are available for MLPA assays. The electrophoresis-based platform requires the use of capillary electrophoresis systems which are available in most institutions; however long oligonucleotides (> 150 nt) in high quality are difficult to synthesize currently. The bead-coupled platform works with half probes (LPO/RPO) less than 100 nucleotides in length, but requires the use of a Luminex system. For the electrophoresis-based platform, probe length increase is achieved either by inserting stuffer sequences between the PCR primer and hybridizing sequences, or by extending the length of hybridizing sequences. By using stuffer sequences, hybridizing sequences have identical length and therefore have a more unified Tm. Since the hybridizing sequences are short, the use of stuffer provides the potential for detecting mutations/polymorphisms that are in close proximity to each other. However, designing stuffer sequences takes time. The stuffer sequences in additional file 3 have been verified in advance so that the union of default PCR primer (used by the commercial MRC-Holland MLPA kits) and stuffer sequences (default left primer + left stuffer; right stuffer + default right primer) are free of secondary structure or significant homology in the human genome. Some workers prefer extending hybridizing sequences to the use of stuffer sequences. However, longer hybridizing sequences are not suitable for short target regions and tend to result in more non-specific binding to the target genome. For the bead-coupled platform, the tag sequence can theoretically be incorporated in either the LPO or RPO. Currently, since anti-tags are linked at their 5' end to the commercially available FlexMAP beads, we recommend inserting the tag sequences in the RPO as illustrated in additional file 2. Thus, only the right PCR primer is in physical proximity to the bead, minimizing any steric hindrance between the PCR product and the bead. Another reason favouring inserting the tag in the RPO is that quite a few tag sequences start with adenosine (additional file 4). These tags will affect probe signal strength if inserted between the left primer and LHS. The commercial FlexMAP tag sequences were not designed specifically for MLPA assays, and some tag sequences, when attached to the default right primer, will form significant secondary structure on their own. For example, the tags corresponding to bead 062 and 071, when attached to the default right primer, have secondary structures that are significant: ΔG = -1.538 and -1.514 respectively (additional file 4). To avoid the use of these tags, the user can insert a short dummy sequence (for example, ACGT) in the input sequence corresponding to that tag (for example, when designing an assay with tags attached to the right primer, input sequence 62 should be input as a dummy sequence, so that tag 062 is consumed by the dummy sequence). Future development of H-MAPD should allow users to use their own stuffer sequences or bead tag sequences. H-MAPD allows users to specify custom PCR primers. However, users should be careful that the union of custom primers to the stuffer/tag sequences may result in secondary structures or significant homology to the human genome, even before specific hybridizing sequences are appended.

It is a challenge to design MLPA probes that can distinguish closely related sequences in the genome. For highly similar but non-identical sequences, H-MAPD will treat them as non-specific matches and is likely to fail the probe set. One can design MLPA probes for highly related sequences by allowing multiple perfect matches to amplify the common identical fragments. Of course the result represents multiple targets, and can not be used to distinguish the highly related but different sequences. A practical solution, as indicated in the MRC-Holland probe design guidelines [4], is to find the exact difference between the sequence of interest and its related sequences, and place the difference at the end of LPO or RPO. A branch of H-MAPD specifically designed for this purpose will be implemented in the future.

## Conclusion

H-MAPD is a web-based MLPA probe design program implemented in Perl-CGI, and hosted on a Linux server. With the increasing application of MLPA in biomedical research and the lack of free probe design software, we hope that H-MAPD will become a valuable tool for automatic MLPA probe generation and selection.

## Availability and requirements

H-MAPD is freely available to non-commercial users at URL . Commercial users should contact the authors. Since the software utilizes the UCSC genome browser and UNAFold, commercial users also need to obtain a license for those programs.

## Abbreviations

MLPA: multiplex ligation-dependent probe amplification; FISH: fluorescent in situ hybridization; UCSC: University of California at Santa Cruz; SNP: single nucleotide polymorphism; LPO: left probe oligo; RPO: right probe oligo; LHS: left hybridizing sequence; RHS: right hybridizing sequence; Tm: melting temperature; ΔG: minimum free energy change; qPCR: quantitative polymerase chain reaction; nt: nucleotide.

## Authors' contributions

JZ implemented the software and web interface, defined score calculation equations, and drafted the manuscript. EH initiated the project, specified the probe design requirements, and modified the manuscript.

## Supplementary Material

Additional file 1 **Diagram of electrophoresis-base MLPA**. Two sequence-tagged half probes are annealed to adjacent sites on the genomic target sequence and ligated using a thermostable DNA ligase. The ligated probes are subsequently amplified with universal primers (one of which is fluorescently labeled) and quantified using electrophoresis. By inserting different-sized stuffer sequence between hybridizing sequence and primer sequence, or by extending the length of the hybridizing sequences, each product has a distinct size, which allows for identification by electrophoresis. The default left and right primers used in H-MAPD are GGGTTCCCTAAGGGTTGGA and TCTAGATTGGATCTTGCTGGCAC, respectively. These are the same primers included in the commercial MRC-Holland MLPA kits. Click here for file

Additional file 2 **Diagram of bead-coupled MLPA**. Similar to electrophoresis-based MLPA, except that the stuffer sequence is replaced by a fixed-length bead tag. Identification of distinct sequences is based on association with a distinct bead. Bead tags can be inserted either between the left primer and the LHS or between the RHS and the right primer. The default left and right primers used in H-MAPD are GGGTTCCCTAAGGGTTGGA and TCTAGATTGGATCTTGCTGGCAC, respectively. These are the same primers included in the commercial MRC-Holland MLPA kits. Click here for file

Additional file 3 **Stuffer sequences**. The stuffer sequences (used in electrophoresis-based MPLA) are from different locations of the Lambda genomic sequence with minor modifications. To ensure that the union of primer and stuffer sequence itself does not fail any of the criteria, firstly all (default left primer GGGTTCCCTAAGGGTTGGA + left stuffer) sequences and (right stuffer + default right primer TCTAGATTGGATCTTGCTGGCAC) sequences were verified to be free of secondary structure at 60°C and 0.35 M Sodium concentration; secondly the maximum Tm of primer and stuffer union sequences to the human genome is verified to be less than 55°C; thirdly no self or inter-probe annealing is detected for all (left primer + left stuffer) and (right stuffer + right primer) sequences. Click here for file

Additional file 4 **FlexMAP bead tag sequences**. FlexMAP bead tag sequences (used in bead-coupled MPLA) are commercially available. ΔG and maximum Tm to the human genome was calculated for (default left primer GGGTTCCCTAAGGGTTGGA + tag) and (tag + default right primer TCTAGATTGGATCTTGCTGGCAC) union sequences. Some of the tag sequences are not suitable for certain MLPA assays. For example, the tag corresponding to bead 062 or bead 071, when attached to the right primer, has a secondary structure that is significant (ΔG = -1.538 and -1.514 respectively). Click here for file

Additional file 5 **Comparison of Tm calculated by RAW and UNAFold**. Melting temperatures of reference sequences mentioned in the MRC-Holland MLPA probe design guidelines, were calculated using two different software, RAW and UNAFold (version 3.5), at 0.35 M Sodium concentration. Tm calculated by RAW is on average 9.1°C higher than that calculated by UNAFold with a standard deviation of 2.8°C. Click here for file
