# Supplementary figures and images for: Human MLPA Probe Design (H-MAPD): a probe design tool for both electrophoresis-based and bead-coupled human multiplex ligation-dependent probe amplification assays
Source: BMC Genomics. 2008 Sep 10;9:407. doi: 10.1186/1471-2164-9-407 (PMC2547856; doi:10.1186/1471-2164-9-407)

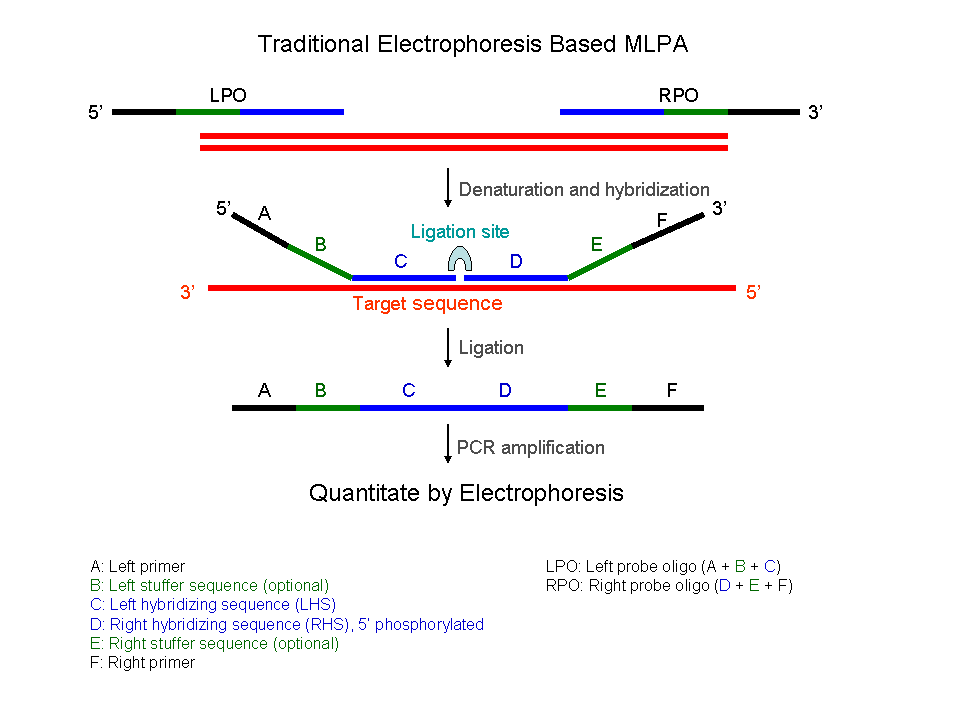

Supplement: Additional file 1 — Diagram of electrophoresis-base MLPA. Two sequence-tagged half probes are annealed to adjacent sites on the genomic target sequence and ligated using a thermostable DNA ligase. The ligated probes are subsequently amplified with universal primers (one of which is fluorescently labeled) and quantified using electrophoresis. By inserting different-sized stuffer sequence between hybridizing sequence and primer sequence, or by extending the length of the hybridizing sequences, each product has a distinct size, which allows for identification by electrophoresis. The default left and right primers used in H-MAPD are GGGTTCCCTAAGGGTTGGA and TCTAGATTGGATCTTGCTGGCAC, respectively. These are the same primers included in the commercial MRC-Holland MLPA kits. [file 1471-2164-9-407-S1.gif]

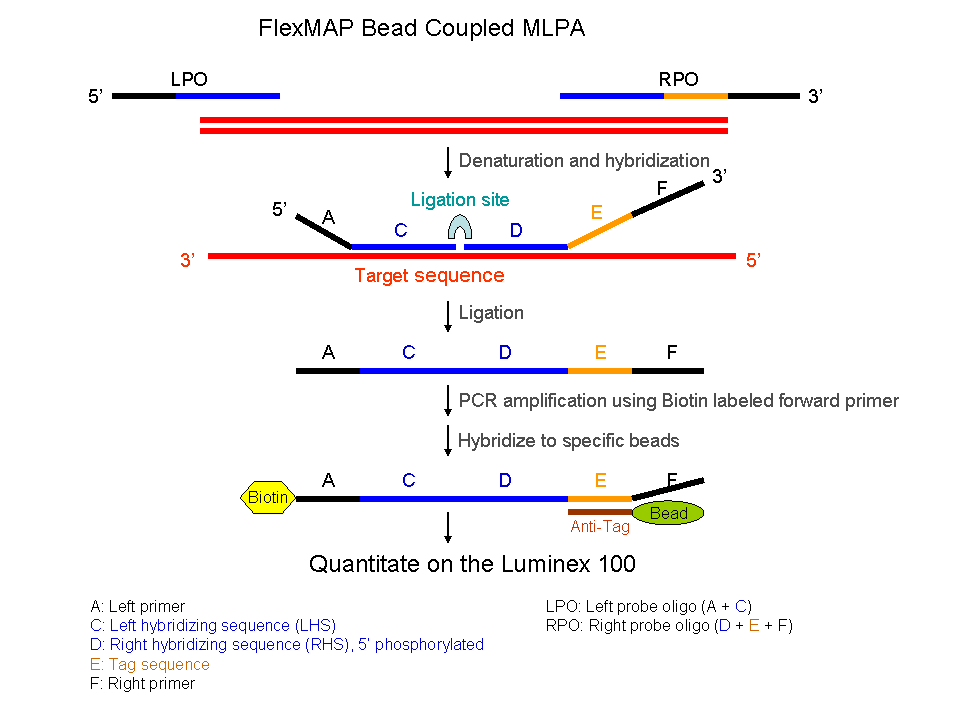

Supplement: Additional file 2 — Diagram of bead-coupled MLPA. Similar to electrophoresis-based MLPA, except that the stuffer sequence is replaced by a fixed-length bead tag. Identification of distinct sequences is based on association with a distinct bead. Bead tags can be inserted either between the left primer and the LHS or between the RHS and the right primer. The default left and right primers used in H-MAPD are GGGTTCCCTAAGGGTTGGA and TCTAGATTGGATCTTGCTGGCAC, respectively. These are the same primers included in the commercial MRC-Holland MLPA kits. [file 1471-2164-9-407-S2.gif]
