# Supplementary material for: Human MLPA Probe Design (H-MAPD): a probe design tool for both electrophoresis-based and bead-coupled human multiplex ligation-dependent probe amplification assays
Source: BMC Genomics. 2008 Sep 10;9:407. doi: 10.1186/1471-2164-9-407 (PMC2547856; doi:10.1186/1471-2164-9-407)
Supplement: Additional file 3 — Stuffer sequences. The stuffer sequences (used in electrophoresis-based MPLA) are from different locations of the Lambda genomic sequence with minor modifications. To ensure that the union of primer and stuffer sequence itself does not fail any of the criteria, firstly all (default left primer GGGTTCCCTAAGGGTTGGA + left stuffer) sequences and (right stuffer + default right primer TCTAGATTGGATCTTGCTGGCAC) sequences were verified to be free of secondary structure at 60°C and 0.35 M Sodium concentration; secondly the maximum Tm of primer and stuffer union sequences to the human genome is verified to be less than 55°C; thirdly no self or inter-probe annealing is detected for all (left primer + left stuffer) and (right stuffer + right primer) sequences. [file 1471-2164-9-407-S3.pdf]

Stuffer Sequences

Increment	Left Stuffer Sequence	Right Stuffer Sequence
4	tg	ac
8	tcaa	aaag
12	taggcc	agcgag
16	tcggcgtt	ttccggaa
20	tgtgaatggg	cggatgctaa
24	tcagcgcaacac	ccttatctggtt
28	taaaaaactaccgt	gaaaagtcggtgga
32	tctggacccgtgatgg	cattctctggttttcg
36	tcatccggtgaagagatt	gagccacctgacagtgtg
40	tgtgggaggcgaaaattggc	gaacgtccggatgctgaagt
44	tgccacgacgatgaacagacgc	tgctgcgtgtggatgaggccat
48	tctgacctttcacatctggacagc	gtacagcccgttcagcacctgggt
52	tgcaggtcgaaaaatgggtggatggc	aggaaagcaatactctgggacacgta
56	tattacgcccgtgccttatccggagagg	atgaatgacgcgacaggaagaacttgac
60	tcggtgagacgtgggaggcgaaaattggcg	aacgtccggatgctgaagtgatggcagagc
64	gagcggaaagagcattattcagcgcccgttcc	tgaccgtgtggcttacctgaccgccggtatcg
68	gactcccagctggaccgctacgaaatgcgcgtat	ggggatgggggccgggtgaggaaagctggctgat
72	gtccaagggaccgagtgaaagtgtggatgcagccct	gttgcccaactttacccgtggcaatgcccgcgcaca
76	gtgttcgatccgaaaggctgggcgctgttccgttcctt	caaagccgtcaaggacaacgtggatacccgtcgtggct
80	gacattgttacactgtggaggagtccatgacgaaagatga	actgattgcccgtctccgctcgctgggtgaacaactgaac
84	gtgagcagtcaggtggcgtgatacgtggtgtttttgatgacc	ctgaaaatatcagctatgccggacagggcgtgcgcgttgaag
88	gtatggtggccagcggctatgactaccggcgcgacgatgatgcg	ggcttgtggagttcagccgatctgacttatgtcattacctatga
92	gcaggaagtcgttaccacctggcccacggagccaatttctcatgct	gaaaacgtggtgtaccggctgtctggtatgtatgagtttgtggtga
96	gatgcgtgatggtggcctgttctccggaggtggacgatgaagaccttc	cgctggaaagtgaaacccggtatggatgtggcttcggtcccttctgta
100	ggtgacgagtgcggttatagcggtccggctgtcgcggatgaatatgacca	gccaacgtccgatatcacgaaggataaatgcagcaaatgcctgagcggtt
104	ggaagtccgcctgctggttcagatacaacgtaacggtggctgggtgacggaa	aaagacatcaccattaagcgcaaaacgacgtcgcagtatctggcctcggtgg
108	gtcgaggattgagctgacgccgggctattttcagataaccgccacgccgcatct	tgccgtttatgacccgacggtacagtttgagttctggttctcggaaaagcagat
112	gacagcgtgaaagcagtgtggactggccgtcaggtacccgtactgtcaccgtgacc	gatgaccatccttttgatcgccagatagtggtgcttccgctgactgacgtttcgag
116	gcacttgcggtgacagtcactcaggcccgtgcggaaggtggacatggtacgtttacgg	tgggctattttcaagtgaaaccgggtacattgccgtcgttgttgtcgggcggggatac
120	gtggttcgccgcgtcgaaaaagagcagcacagtgatgaccggggaggatacgtttcacta	tgagagcctgcgtggacgttatgtgagcgtgatggccggaccggttttacaaatcagtaa
124	cgtaagacggaaatcactcccgggtatatgaaagagacgaccactgccagggacgaaagtgc	aatgcggcatacctcagtggcgtggagtgcaggtatacagattaatccggcagcgtccgtcg
128	ctgatgaagccgggcgttacagcatggatgtggagtacggtcagtacagtgtcatcctgcaggt	tgacggttttccaccatcgcacgccgggaccatcaccgtgtatgaagattcacaaccggggacg
132	ctggaagtgcggtatcagcatcgcagagcaaaagtgcggcagaagcggcggcaatacgtgcaaaaa	attcggcaaaacgtgcagaagatatagcttcagctgtcgcgcttgaggatgcggacacaacgagaa
136	ctcggaaaacaccgtcaaaaacattgcataattctatattgtgaggcttgcataatggcattcagaat	gagtgaacaaccacggaccataaaaatttctgctggccggaactaatgaatttattggtgaaggtga
140	cctggaagcctggaagaagtatcgggtgttgctgaaccgtgttgatacatcaactgcacctgatattgag	tggcctgctgtccctgttatggagtaatcggttttgtgatatgccgcagaaagcttgtatgaaataacgt
144	cttcaggagaataatggaagttctatgactcaattgttcatagtgtttacatcaccgccaattgcttttaag	actgaacgcatgaaatatggtttttcgtatgttttgagcttgctgttgatatttctaaagtcggttttttt
148	cattttgattattatttgaatcaattccaattacctgaagtctttcatctataattggcattgtatgtattgg	tttattggagtagatgcttgctaatctatgtattggtttattggagtagatgcttgcttttctgacgcatagct
152	caattaatgtttgaatgtgataaccgtcctttaaaaaagtcgtttctgcaagcttggctgtatagtcaactaactc	ttctgtcgaagtgatatttttaggtatctaccagttttagacgctctttaatatcttcaggaattattttattgtc
156	ctaaataagttattctcctggcttcatcaaataaagagtcgaatgatgttggcgaaatcacatcgtcacccattggat	tgtttatttgtatgccaagagagttacagcagttatacattctgccatagattatagctaaggcatgtaataattcgt
160	catgttactgttcttgcggtttggaggaattgattcaaattcaagcgaaataattcagggtcaaaatatgtatcaatgca	gcatttgagcaagtgcgataaatctttaagtcttctttcccatggtttttagtcataaaactctccattttgataggtt
164	cacgtcgtatgcaggaatgtgctgcggctggctggtgaacttccgatagtgcgggtgttgaatgatttccagttgctaccga	ttttacatattaattgcacgattttacatattaattcgatgagagaatttgtaccacctcccaccgaccatctatgactgta
168	caccggattttgtaaaaacagccctcctcatataaaaagtattcgttcacttccgataagcgtcgtaattttctatctttcatc	atattctagatccctctgaaaaaatcttccgagtttgctaggcactgatacataactgttttccaataattcgggaagtcattc
172	cgtttcagatttcgttcaataaattctgactgtagctgctgatagcttgcggttgaactatatttccttataacttttacgataga	gtttctttgagtaacttcactcaagtgcttccctgcctccaaacgatacctgttagcaatatttaatagcttgaaatgatgaagag
176	cgaaatttgcatatacccattgctcacgaaaaaaaatgtccttgtcgatatagggatgaatcgcttggtgtacctcatctactgcgaa	cacaaaatggacattttcacctgatgaaacaagcatgtcatcgtaatatgttctagcgggtttgtttttatctcggagattattttc
180	cctttgtcagcttaccaactactaagcttgtaggctcaagagcgtgtgtcctgtcgtaggtaaataactgacctgtcgagcttaatattc	tatattgttgatttttagaatggtttcataagaaagctctgaatcaacggactgcgataataagtggtggtatccagaatttgtcactt
184	cgtaggactttccacatgcaggattttggaacctcttgcagtactactggggaatgagttgcaattattgctacaccattgcgtgcatcgag	taagtcgcaagcagatatttctggatattgtcataaaagaatttactgaatttatcatcgtccacttgaatctgtggttcattacgtcttaa
188	ctgagatcgcctagtgattttaaactattgctggcagcattcttgagtccaatataaaagtattgtgtaccttttgctgggtcaggttgttctt	taggagaggtatagttaaatcactgaatccgggagcactttttctattaaatgaaaagtggaaatctcagatatctggcaaaccattatacaca
192	ctctgaatagctttaagaaggttatgtttaaaaccatcgcttaatttcgtgagattaacatagtagtcaatgctttcacctaaggaaaaaaacatt	tcagtaacaaaggaatagtcattccaaccatctgctcgtaggaatcggttattttttttctactgcaggaatatacccgcctctttcaataacacta
196	ctcaatttatttggcggcaacacaggatctctcttttaagttactctctattacatacgttttccatctaaaaattagtagtattgaacttaacgggg	catgcttccttttggataacccactgttattcatgttgcatggtgcactgtttataccaacgatatagtctattaatgcatatatagtatcgccgaac
200	cacttcgtagccatttttcataagtgttaacttccgctcctcgctcataacagacattcactacagttatggcggaaaggtatgcatgctgggtgtgggg	caagaagacaaaaatcaccttgcgctaatgctctgttacaggtcactaataccatctaagtagttgattcatagtgactgcatatgttgtgttttacagt

The stuffer sequences are from different locations of Lambda genomic sequence with minor modifications

All (default left primer + left stuffer) sequences and (right stuffer + default right primer) sequences are free of secondary structures at 60 ºC and 0.35 M Sodium concentration (verified by free energy calculation)

The maximum Tm to hg18 is less than 55 ºC for all (default left primer + left stuffer) sequences and (right stuffer + default right primer) sequences (verified by BLAT search and Tm calculation)

No self or inter-probe annealing is detected for all (default left primer + left stuffer) sequences and (right stuffer + default right primer) sequences (verified by Tm calculation)
