# Supplementary material for: Human MLPA Probe Design (H-MAPD): a probe design tool for both electrophoresis-based and bead-coupled human multiplex ligation-dependent probe amplification assays
Source: BMC Genomics. 2008 Sep 10;9:407. doi: 10.1186/1471-2164-9-407 (PMC2547856; doi:10.1186/1471-2164-9-407)
Supplement: Additional file 4 — FlexMAP bead tag sequences. FlexMAP bead tag sequences (used in bead-coupled MPLA) are commercially available. ΔG and maximum Tm to the human genome was calculated for (default left primer GGGTTCCCTAAGGGTTGGA + tag) and (tag + default right primer TCTAGATTGGATCTTGCTGGCAC) union sequences. Some of the tag sequences are not suitable for certain MLPA assays. For example, the tag corresponding to bead 062 or bead 071, when attached to the right primer, has a secondary structure that is significant (ΔG = -1.538 and -1.514 respectively). [file 1471-2164-9-407-S4.pdf]

FlexMAP Bead Tag Sequences (Commercial)

Bead	Tag (appended to primer)	Appended to left primer		Appended to right primer	
		ΔG	Max. Tm to hg18	ΔG	Max. Tm to hg18
001	ctttaatctcaatcaatacaaatc	0.988	N/A	0.866	40.0
002	ctttatcaatacatactacaatca	0.988	N/A	-0.043	41.0
003	tacactttatcaaatcttacaatc	0.429	N/A	0.41	N/A
004	tacattaccaataatcttcaaatc	0.446	N/A	0.866	37.0
005	caattcaaatcacaataatcaatc	0.988	N/A	0.648	N/A
006	tcaacaatcttttacaatcaaatc	0.988	N/A	0.597	41.2
007	caattcatttaccaatttaccaat	0.988	N/A	-0.79	42.0
008	aatccttttacattcattacttac	-0.391	N/A	0.992	N/A
009	taatcttctatatcaacatcttac	0.988	N/A	0.992	N/A
010	atcatacatacatacaaatctaca	0.988	N/A	0.688	N/A
011	tacaaatcatcaatcactttaatc	0.086	36.7	0.992	N/A
012	tacactttctttctttctttcttt	0.429	49.0	0.992	N/A
013	caataaactatacttcttcactaa	0.988	N/A	0.992	39.0
014	ctactatacatcttactatacttt	0.988	N/A	0.992	40.8
015	atacttcattcattcatcaattca	0.988	42.5	0.992	46.9
016	aatcaatcttcattcaaatcatca	0.988	N/A	0.732	41.5
017	ctttaatcctttatcactttatca	0.712	N/A	0.977	41.3
018	tcaaaatctcaaatactcaaatca	0.988	N/A	0.413	39.5
019	tcaatcaattacttactcaaatac	0.988	N/A	0.992	39.2
020	cttttacaatacttcaatacaatc	0.988	42.9	-0.387	38.4
021	aatcctttctttaatctcaaatca	-0.391	N/A	0.413	N/A
022	aatccttttactcaattcaatca	-0.391	N/A	-0.22	N/A
023	ttcaatcattcaaatctcaacttt	0.988	N/A	0.992	42.3
024	tcaattaccttttcaatacaatac	0.988	N/A	0.992	42.0
025	cttttcaattacttcaaatcttca	0.988	N/A	0.992	N/A
026	ttactcaaaatctacactttttca	0.988	N/A	0.992	N/A
027	cttttcaaatcaatactcaacttt	0.988	N/A	0.992	N/A
028	ctacaaacaaacaaacattatcaa	0.988	N/A	0.992	34.9
029	aatcttactacaaatcctttcttt	0.988	N/A	0.992	N/A
030	ttacctttatacctttctttttac	0.8	N/A	0.992	N/A
031	ttcacttttcaatcaactttaatc	0.988	43.2	0.992	43.2
032	attattcacttcaaactaatctac	0.988	N/A	0.59	N/A
033	tcaattacttcactttaatccttt	0.988	N/A	0.881	N/A
034	tcattcatatacataccaattcat	0.988	N/A	0.284	N/A
035	caatttcatcattcattcatttca	0.988	40.5	0.992	41.4
036	caattcatttcattcacaatcaat	0.988	N/A	0.992	47.1
037	cttttcatcttttcatctttcaat	0.988	40.6	0.992	41.3
038	tcaatcattacacttttcaacaat	0.988	37.1	0.992	N/A
039	tacacaatcttttcattacatcat	0.446	N/A	0.992	39.8
040	ctttctacattattcacaacatta	0.988	N/A	0.992	N/A
041	ttactacacaatatactcatcaat	0.988	N/A	0.992	41.7
042	ctatcttcatatttcactataaac	0.988	N/A	0.992	28.9
043	ctttcaattacaatactcattaca	0.988	N/A	0.992	N/A
044	tcatttaccaatctttctttatac	0.988	43.3	-0.504	N/A
045	tcatttcacaattcaattactcaa	0.988	N/A	0.992	38.1
046	tacatcaacaattcattcaataca	0.397	N/A	0.992	N/A
047	cttctcattaacttacttcataat	0.988	N/A	0.992	N/A
048	aaacaaacttcacatctcaataat	0.374	N/A	0.992	N/A
049	tcatcaatctttcaatttacttac	0.988	N/A	0.992	N/A
050	caatataccaatatcatcatttac	0.988	N/A	0.939	N/A

051	tcatttcaatcaatcatcaacaat	0.988	47.0	0.992	N/A
052	tcaatcatctttatacttcacaat	0.988	N/A	0.992	47.2
053	taattatacatctcatcttctaca	0.988	24.7	0.992	N/A
054	ctttttcaatcactttcaattcat	0.988	N/A	0.992	N/A
055	tatatacacttctcaataactaac	0.988	41.8	0.992	N/A
056	caatttactcatatacatcacttt	0.988	31.8	0.992	N/A
057	caatatcatcatctttatcattac	0.988	37.6	0.992	37.6
058	ctactaattcattaacattactac	0.988	N/A	0.992	N/A
059	tcatcaatcaatctttttcacttt	0.988	N/A	0.992	39.3
060	aatctacaaatccaataatctcat	0.921	N/A	-0.131	N/A
061	aatcttaccaattcataatcttca	0.988	N/A	0.928	N/A
062	tcaatcataatctcataatccaat	0.988	N/A	-1.538	32.6
063	ctacttcatatactttatactaca	0.988	N/A	0.992	N/A
064	ctacatattcaaattactacttac	0.988	N/A	0.992	N/A
065	cttttcatcaataatcttaccttt	0.988	N/A	0.992	N/A
066	taacattacaactatactatctac	0.988	N/A	0.902	N/A
067	tcatttactcaacaattacaaatc	0.988	N/A	0.866	N/A
068	tcataatctcaacaatctttcttt	0.988	32.7	0.992	45.9
069	ctataaacatattacattcacatc	0.988	N/A	0.992	N/A
070	ataccaataatccaattcatatca	0.608	N/A	-0.142	N/A
071	atcattacaatccaatcaattcat	0.171	36.6	-1.514	N/A
072	tcatttacctttaatccaataatc	0.988	44.4	-0.453	N/A
073	atcaaatctcatcaattcaacaat	0.988	52.4	0.992	N/A
074	tacacatcttacaaactaatttca	0.446	40.2	0.992	N/A
075	aatcatacctttcaatcttttaca	0.988	N/A	0.992	N/A
076	aatctaacaaactcatctaaatac	0.988	N/A	0.992	N/A
077	caattaactacatacaatacatac	0.988	N/A	0.992	N/A
078	ctatctatctaactatctatatca	0.988	40.7	0.977	43.3
079	ttcataactacaatacatcatcat	0.988	N/A	0.992	33.8
080	ctaactaacaataatctaactaac	0.988	N/A	0.992	N/A
081	ctttaatctacactttctaacaat	0.988	41.4	0.992	N/A
082	tacatacactaataacatactcat	0.446	N/A	0.992	N/A
083	atacaatctaacttcactattaca	0.988	N/A	0.992	N/A
084	tcaactaactaatcatctatcaat	0.988	N/A	0.992	N/A
085	atactacatcataatcaaacatca	0.988	42.7	0.732	41.3
086	ctaattactaacatcactaacaat	0.988	N/A	0.992	N/A
087	aaactaacatcaatacttacatca	0.644	N/A	0.732	N/A
088	ttacttcactttctatttacaatc	0.988	43.0	0.41	N/A
089	tatactatcaactcaacaacatat	0.988	N/A	0.992	39.6
090	ctaaatacttcacaattcatctaa	0.988	N/A	0.657	N/A
091	ttcataacatcaatcataacttac	0.988	N/A	0.992	N/A
092	ctattacactttaaacatcaatac	0.988	N/A	0.992	N/A
093	ctttctattcatctaaatacaaac	0.988	N/A	0.992	N/A
094	ctttctatctttctactcaataat	0.988	34.5	0.992	N/A
095	tacactttaaacttactacactaa	0.429	31.7	0.992	N/A
096	atactaactcaactaactttaaac	0.988	N/A	0.992	N/A
097	aatctcataatctacatacactat	0.988	N/A	0.992	N/A
098	aatcatactcaactaatcattcaa	0.988	N/A	0.96	N/A
099	aatctacactaacaatttcataac	0.988	N/A	0.992	N/A
100	ctatctttaaactacaaatctaac	0.988	N/A	0.61	N/A

N/A: no significant homology to hg18
