# Supplementary material for: Human MLPA Probe Design (H-MAPD): a probe design tool for both electrophoresis-based and bead-coupled human multiplex ligation-dependent probe amplification assays
Source: BMC Genomics. 2008 Sep 10;9:407. doi: 10.1186/1471-2164-9-407 (PMC2547856; doi:10.1186/1471-2164-9-407)
Supplement: Additional file 5 — Comparison of Tm calculated by RAW and UNAFold. Melting temperatures of reference sequences mentioned in the MRC-Holland MLPA probe design guidelines, were calculated using two different software, RAW and UNAFold (version 3.5), at 0.35 M Sodium concentration. Tm calculated by RAW is on average 9.1°C higher than that calculated by UNAFold with a standard deviation of 2.8°C. [file 1471-2164-9-407-S5.pdf]

Name	Sequence	RAW Tm (ºC)	UNAFold Tm (ºC)
ADAM-D07 LHS	GCAGGACCCGGAAGTACCTGGAACTGT	77.6	65.9
ADAM-D07 RHS	ACATTGTGGCAGACCACACCCTGGTGAGGAGAGACC	85.4	73.5
DPYD-D023 LHS	GAGATGATAGGATAGATCCTGGTTACCACTCTT	71.0	64.1
DPYD-D023 RHS	TTGCTGTGCACATACGGGCTCTGACTGGTTTTAATAGTCAC	79.4	71.4
C21ORF63-D0 LHS	GATGCTGTGGCCCACTTTAAAACAA	70.2	59.3
C21ORF63-D0 RHS	AGCCCAATTATTAGCGCTCGGCGGCT	72.2	67.1
